# Identification of Differentially Expressed miRNAs in Colorado Potato Beetles (*Leptinotarsa decemlineata* (Say)) Exposed to Imidacloprid

**DOI:** 10.3390/ijms18122728

**Published:** 2017-12-16

**Authors:** Mathieu D. Morin, Pierre J. Lyons, Nicolas Crapoulet, Sébastien Boquel, Pier Jr Morin

**Affiliations:** 1 Department of Chemistry and Biochemistry, University of Moncton, 18 Antonine-Maillet Avenue, Moncton, NB E1A 3E9, Canada; mathieu.morin@umoncton.ca; 2 Atlantic Cancer Research Institute, Pavillon Hôtel-Dieu 35 Providence Street, Moncton, NB E1C 8X3, Canada; pierrelyons@gmail.com (P.J.L.); nicolas.crapoulet@vitalitenb.ca (N.C.); 3 Fredericton Research and Development Centre, Agriculture and Agri-Food Canada, 850 Lincoln Road, Fredericton, NB E3B 4Z7, Canada; sebastien.boquel@agr.gc.ca

**Keywords:** microRNAs, cold hardiness, Colorado potato beetles, imidacloprid, next-generation sequencing

## Abstract

The Colorado potato beetle (*Leptinotarsa decemlineata* (Say)) is a significant pest of potato plants that has been controlled for more than two decades by neonicotinoid imidacloprid. *L. decemlineata* can develop resistance to this agent even though the molecular mechanisms underlying this resistance are not well characterized. MicroRNAs (miRNAs) are short ribonucleic acids that have been linked to response to various insecticides in several insect models. Unfortunately, the information is lacking regarding differentially expressed miRNAs following imidacloprid treatment in *L. decemlineata*. In this study, next-generation sequencing and quantitative real-time polymerase chain reaction (qRT-PCR) were used to identify modulated miRNAs in imidacloprid-treated versus untreated *L. decemlineata*. This approach identified 33 differentially expressed miRNAs between the two experimental conditions. Of interest, miR-282 and miR-989, miRNAs previously shown to be modulated by imidacloprid in other insects, and miR-100, a miRNA associated with regulation of cytochrome P450 expression, were significantly modulated in imidacloprid-treated beetles. Overall, this work presents the first report of a miRNA signature associated with imidacloprid exposure in *L. decemlineata* using a high-throughput approach. It also reveals interesting miRNA candidates that potentially underly imidacloprid response in this insect pest.

## 1. Introduction

The Colorado potato beetle (CPB) (*Leptinotarsa decemlineata* (Say)) is a significant insect pest harming potato crops worldwide [1]. CPBs are often considered to be the primary insect associated with potato plant defoliation [2], leading to up to 75% of foliage consumption and impacting revenues for growers [3,4]. Pest control strategies targeting CPBs typically involve pesticides, even though resistance against a variety of such compounds, including spinosad, thiamethoxam, and deltamethrin, has been observed [5,6,7]. Neonicotinoids, which target the nicotinic acetylcholine receptors (nAChRs), are a class of insecticides that are used extensively against CPBs. The neonicotinoid imidacloprid (1-(6-chloro-3-pyridylmethyl)-*N*-nitroimidazolidin-2-ylideneamine) was registered for such a purpose over 20 years ago, and was initially successful in managing CPB populations [6]. Unfortunately, early studies reported adult CPBs exhibiting as much as a 155-fold increase in imidacloprid resistance in select populations [8]. Efforts have been deployed in recent years to understand the molecular players associated with imidacloprid resistance in CPBs. Multiple studies have notably highlighted the differential expression of cytochrome P450s in imidacloprid-treated CPBs [9,10], positioning these enzymes as targets that could sensitize CBPs to this agent. Nevertheless, the complete molecular picture associated with imidacloprid resistance in CPBs remains incompletely characterized.

MicroRNAs (miRNAs) are short conserved non-coding RNAs capable of regulating the expression of multiple target mRNA transcripts. These molecules are involved in the response to various stresses including starvation, anoxia, and freezing [11,12]. Previous work has also revealed miRNA modulation in insects following exposure to different chemicals including pyrethroids, chlorantraniliprole and fenpropathrin [13,14,15]. In addition, recent studies have highlighted the likely regulation of pyrethroid resistance in the mosquito *Culex pipiens pallens* (L.) by miRNAs [16,17]. These results thus support the potential involvement of miRNAs in insecticide resistance. Unfortunately, data is currently lacking regarding a signature of differentially expressed miRNAs in response to imidacloprid treatment in CPBs.

The current study was undertaken to characterize the miRNA footprint observed in CPBs following imidacloprid exposure, and to identify novel miRNA-associated molecular leads that could be utilized in the development of alternative strategies to control CPBs using an approach relying on next-generation sequencing and qRT-PCR. The miRNA target prediction was also performed using predictive tools to better assess the likely consequences of these imidacloprid-associated miRNAs. Transcript targets linked to transcriptional regulation and glucose metabolism were observed and are further discussed.

## 2. Results

### 2.1. Small RNA Sequence Analysis

High-throughput sequencing resulted in 59,406,387 reads among all samples. Low quality reads were discarded. Reads ranging from 16 to 60 nucleotides were conserved for analysis. Small RNA libraries displayed comparable read distribution profiles in the 16 and 24 nucleotides region, with maximum peaks observed at 21 nucleotides. This approach resulted in 22,534,030 and 13,763,160 total reads for control and imidacloprid-treated insects, respectively (Table 1). Within these reads, 8,825,610 (control) and 6,211,853 (imidacloprid) unique reads were obtained. MiRBase was utilized to identify the small RNAs of interest in *L. decemlineata* samples using red flour beetle *Tribolium castaneum* reference databases [18]. The miRNAs were annotated using sRNAbench in sRNAtoolbox [19]. Small RNAs unique reads were mapped to miRNAs (26,447 for control and 24,221 for imidacloprid-treated). SnRNAs (4735 for control and 3616 for imidacloprid-treated), snRNAs (4705 for control and 2937 for imidacloprid-treated) and tRNAs (40,144 for control and 22,115 for imidacloprid-treated) were also observed.

### 2.2. miRNA Expression in Control and Imidacloprid-Treated L. decemlineata by High-Throughput Sequencing

The most frequent miRNAs detected with this approach were miR-14-3p (95,802 reads in control and 81,633 reads in imidacloprid-exposed insects) and miR-8-3p (68,314 reads in control and 79,134 reads in imidacloprid-exposed insects) (Table 2). Based on next-generation sequencing results, log2 ratios of miRNA expression in control and imidacloprid-exposed *L. decemlineata* demonstrated 33 differentially expressed miRNAs. A total of 14 upregulated and 19 downregulated miRNAs displaying absolute log2 fold-changes greater than 0.3 (*p* < 0.05, *n* = 3) were identified (Table 3).

### 2.3. qRT-PCR Quantification of Selected miRNAs

**Figure 1:**
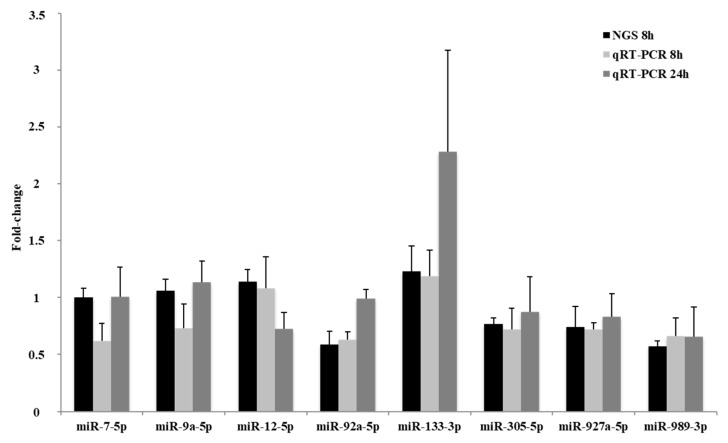
Next-generation sequencing (NGS) and quantitative real-time polymerase chain reaction (qRT-PCR) expression data of select miRNAs. Expression of eight miRNAs quantified in control and imidacloprid-treated *L. decemlineata*. Data for qRT-PCR is normalized to transcript levels (mean ± SEM, *n* = 3) and NGS levels represent fold-changes in normalized read counts (mean ± SEM, *n* = 3).

Eight miRNAs were further amplified and quantified by qRT-PCR. These miRNAs displayed reduced, elevated or stable levels in insects treated with imidacloprid for 8 h when compared with control insects using deep sequencing. Fold-changes were compared with expression results measured by next-generation sequencing (Figure 1). MiR-92a-5p, miR-133-3p, miR-305-5p, miR-927a-5p and miR-989-3p displayed substantial modulation in imidacloprid-treated versus untreated insects as measured by high-throughput sequencing, with 0.59-fold, 1.23-fold, 0.77-fold, 0.74-fold and 0.57-fold variations, respectively. These changes were comparable to the ones obtained by qRT-PCR. The miR-12-5p levels were also comparable when both methods were used to quantify its expression. MiR-7-5p and miR-9a-5p, stable miRNAs in imidacloprid-treated insects as measured by next-generation sequencing, displayed reduced levels (0.62-fold and 0.73-fold, respectively) when measured by qRT-PCR. Assessment of miRNA expression in insects treated with imidacloprid for 24 h was also performed by qRT-PCR (Figure 1). Differential expression of miR-7-5p, miR-9a-5p, miR-12-5p, miR-92a-5p, and miR-133-5p was observed between insects submitted to imidacloprid treatments of varying lengths. Levels of miR-305-5p, miR-927a-5p and miR-989-3p remained stable between the two treatment groups.

### 2.4. Prediction of miRNA Targets and Functional Classification

Target prediction tools were able to identify transcripts potentially regulated by 17 *L. decemlineata* imidacloprid-associated miRNAs. Targets were assessed for the following miRNAs: miR-282-5p, miR-1000-5p, miR-193-3p, miR-124-3p, miR-970-3p, let-7-5p, miR-1-3p, miR-133-3p, miR-263b-5p, miR-92a-3p, miR-305-5p, miR-927a-5p, miR-9c-5p, miR-316-5p, miR-100-5p, miR-315-5p and miR-989-3p (Table 4). Common targets are underlined. Biological processes with predicted transcripts are presented in Table 5. Several target transcripts were associated with transcriptional regulation, whilst others were linked to glucose metabolism.

## 3. Discussion

The molecular changes underlying neonicotinoid resistance in insects have been investigated with great interest in recent years. Work conducted on various insect models treated with imidacloprid has revealed targets potentially involved in the response to this agent. These targets include select cytochrome P450s, ATP-binding cassette (ABC) transporters, and nAChRs [20,21,22]. Numerous examples exist that highlight the regulation of these imidacloprid-relevant targets by miRNAs [23,24]. While recent studies have started to associate miRNAs with insecticide resistance in various insects [14,25,26], no work so far has attempted to characterize modulated miRNAs in imidacloprid-treated CPBs. Using a high-throughput sequencing approach, the current study reveals a signature of differentially expressed miRNAs in CPBs exposed to this neonicotinoid.

The information is sparse regarding the molecular changes associated with imidacloprid response in insects. Pioneering work performed on beehives submitted to low levels of imidacloprid revealed a set of differentially expressed miRNAs in larvae gathered from imidacloprid-exposed versus unexposed hives [27]. The present study also identified similar changes in expression levels of select miRNAs, including miR-282 upregulation and miR-989 downregulation, in imidacloprid-treated CPBs. MiR-989 has been linked with key physiological processes, as *Drosophila melanogaster* (Meigen) miR-989 mutants notably exhibit impaired border cell migration [28], and *Bombyx mori* (L.) strongly upregulates miR-989 during the pupal phases [29]. It is interesting to note that levels of miR-989-3p, like miR-305-5p and miR-927a-5p, remained unchanged in insects treated with imidacloprid for different lengths of time, strengthening its potential involvement in response to this insecticide. MiR-282, on the other hand, can influence *D. melanogaster* viability and longevity by targeting the adenylate cyclase rutabaga [30]. While modulation of miR-282 and miR-989 in imidacloprid-treated CPBs presented here supports their probable role in response to this pesticide, additional work is required to fully delineate their functions in insects exposed to imidacloprid.

Several reports have highlighted the upregulation of cytochrome P450s in CPBs treated with imidacloprid. A high-throughput sequencing approach performed in imidacloprid-sensitive and imidacloprid-resistant CPBs revealed that transcripts for 41 cytochrome P450s were more than 2-fold upregulated in pesticide-exposed insects. In addition, cytochrome P450 activity was increased in the resistant beetles [10]. Gene expression analysis performed on CPB populations susceptible or resistant to imidacloprid reported differential cytochrome P450 levels. This further strengthens an underlying role for these enzymes in imidacloprid resistance [9]. Several studies have reported miRNA-mediated regulation of cytochrome P450 expression. Recent work conducted in the tobacco aphid *Myzus persicae nicotianae* (Blackman) showed that expression of CYP6CY3, a cytochrome P450 previously associated with neonicotinoid resistance [31], was regulated by miR-100 [32]. The present study highlighted downregulation of miR-100-5p levels in imidacloprid-treated CPBs, supporting a potential role for the miR-100-cytochrome P450 axis in the imidacloprid response.

Functional annotation was undertaken using the predicted targets of differentially expressed miRNAs following imidacloprid exposure in order to better characterize the potential impact of this compound in CPBs (Table 5). This approach notably highlighted gene transcription as a likely process affected by the modulated miRNAs. Some miRNAs, such as miR-92a-3p and miR-927a-5p, were predicted to impact key players involved in transcriptional regulation, including the cAMP response element binding (Creb) transcription factor and the ecdysone receptor (EcR). Previous work has reported that Creb levels were differentially expressed in honey bees (*Apis mellifera carnica* (Pollmann)) exposed to various neonicotinoids [33], supporting a potential role for Creb in the response to imidacloprid. Several studies have investigated the effect of imidacloprid treatment on gene expression in multiple insect models. A microarray-based approach performed in the mosquito *Aedes aegypti* (L.) revealed 344 and 108 differentially expressed genes in imidacloprid-resistant larvae and adults, respectively, when compared to their imidacloprid-sensitive counterparts [34]. In addition, a high-throughput sequencing approach in *D. melanogaster* that were sensitive or resistant to the same agent highlighted 357 transcripts that were either upregulated or downregulated in imidacloprid-resistant flies [35]. Multiple transcript targets associated with imidacloprid-modulated miRNAs were also linked to glucose metabolism. Previous reports have linked imidacloprid treatment with modulation of various targets involved in glucose homeostasis. Honey bee larvae exposed to imidacloprid exhibited increased transcript levels of multiple genes involved in the glycolytic and gluconeogenic pathways. Transcript levels of phosphoenolpyruvate carboxykinase (PEPCK), a rate-limiting enzyme for the latter cascade, were notably upregulated in imidacloprid-exposed larvae [27]. In addition, a study performed on Madagascar hissing cockroaches (*Gromphadorhina portentosa* (Schaum)) showed variations in glucose uptake and carbohydrate metabolism in cockroaches exposed to imidacloprid [36]. These leads generate potential processes via which the identified miRNAs can influence imidacloprid response. However, further characterization of the link that exists between these processes and imidacloprid-associated miRNAs is envisioned. Ultimately, such transcripts could be targeted by diverse means including RNA interference (RNAi)-based approaches that form part of a strategy aimed at influencing imidacloprid response in CPBs. Such approaches are currently under investigation in various pests including CPBs [37].

In conclusion, the present study revealed a set of modulated miRNAs in CPBs following exposure to the neonicotinoid imidacloprid, and is aligned with recent work aimed at better understanding the functions associated with miRNAs in these insects [38]. These results present the first example of a miRNA signature in *L. decemlineata* treated with this agent. Assessment of miRNA expression following different imidacloprid treatment durations also revealed varying levels of select miRNAs in short- versus long-term insecticide exposures. This warrants further investigation of early and late molecular changes associated with response to this agent. Predicted miRNA transcript targets further suggest that differential miRNA expression could impact molecular players involved in key processes. It is important to note that while the miRNA changes uncovered in this study generated valuable information regarding miRNAs underlying insect response to imidacloprid, future work is needed to investigate miRNA signatures observed in response to additional experimental conditions. These conditions include repeated topical imidacloprid exposures, usage of a diet comprised of imidacloprid-treated potato leaves, or field CPBs naturally exposed to this agent and exhibiting various degrees of tolerance against imidacloprid. Further investigations will be undertaken to examine the expression status of enzymes such as cytochrome P450s in CPB populations that do or do not respond to imidacloprid. Overall, this work provides novel information on a potential role for miRNAs in imidacloprid response and resistance, as well as contributing to the growing knowledge of the molecular levers underlying the response to this agent in Colorado potato beetles.

## 4. Materials and Methods

### 4.1. Insect Collection and Treatment

Adult Colorado potato beetles that had overwintered were collected in nursery potato fields at the Fredericton Research and Development Centre in Fredericton (NB, Canada) in June 2016. Potato fields where collection occurred were not treated with any types of insecticides. This nevertheless cannot rule out the potential exposure of beetles to insecticides in years prior to the study, nor exposure attributable to insect migration. Beetles were placed in plastic containers (50 per) containing potato leaves, and closed using a lid with insect screening for ventilation. Insects were subsequently transported to the Université de Moncton (Moncton, NB, Canada). A group of 30 insects was placed in an incubator (Thermo Fisher Scientific, Waltham, MA, USA) set at 25 °C for 5 days under 16L:8D cycles. Insects were provided with potato plants. Following acclimation, 5 µL (0.5 µg) of analytical grade imidacloprid (100 µg/mL in acetonitrile, Sigma-Aldrich, St. Louis, MO, USA) was applied topically on the abdomen of 15 beetles, as previously described [10]. A volume of 5 µL acetonitrile was applied on 15 beetles in parallel, which were used as controls. Imidacloprid-treated and control insects were returned to the incubator for 8 h. A similar experimental protocol was undertaken where insects were treated with imidacloprid for 24 h. Insects were active after the incubation period and prior to storage. A parallel bioassay performed on insects treated with increasing imidacloprid doses (0.05 µg to 5.0 µg) and not destined for high-throughput sequencing highlighted a marked impact on insect activity, albeit not viability, at maximum doses of 2.5 µg and 5.0 µg of imidacloprid (LD_50_ >5.0 µg/insect). All insects destined for sequencing were ultimately placed rapidly in liquid nitrogen and stored at −80 °C until RNA isolation.

### 4.2. Small RNA Isolation

Small RNA fractions were isolated from control and imidacloprid-treated *L. decemlineata* using the miRVana miRNA Isolation Kit (Thermo Fisher Scientific, Waltham, MA, USA) as per the manufacturer’s instructions and as described previously [39]. Each isolate was prepared using two *L. decemlineata*. RNA isolates were generated in triplicates. The quality and integrity of small RNA fractions were confirmed using the High Sensitivity RNA ScreenTape on an Agilent TapeStation 2200 instrument (Agilent Technologies (Santa Clara, CA, USA)).

### 4.3. Small RNA Library Construction and Sequencing

Small RNA libraries were prepared and sequenced following Ion Torrent protocols and as reported previously [40]. Samples were loaded on an Ion PI Chip v2 and sequenced with an Ion Proton Sequencer (Thermo Fisher Scientific, Waltham, MA, USA). Three control and three imidacloprid-treated libraries were constructed. FASTQ files were generated for each sample and adaptor sequences were trimmed. Reads with less than 16 and more than 60 nucleotides were discarded. Reads with Q scores of less than 20 were not included. Small RNA annotation was performed using the sRNAbench tool from sRNAtoolbox. Red flour beetle *Tribolium castaneum* was used as a reference [19]. The number of permitted mismatches when mapping to mature miRNAs was set to 3. Default values were used for the remaining parameters. Annotated sequences having less than 10 normalized read counts in either control or insecticide-treated samples were discarded, and log2 fold-changes were generated.

### 4.4. cDNA Synthesis

cDNA was synthetized as described earlier [41]. *L. decemlineata* miRNA sequences obtained via next-generation sequencing were used to design stem-loop primers to amplify miR-1-3p, miR-7-5p, miR-9a-5p, miR-12-5p, miR-92a-5p, miR-133-3p, miR-305-5p, miR-927a-5p and miR-989-3p (Table 6). A volume containing 0.5–1 µg of small RNA was combined with 5 μL of 300 nM of miRNA-specific stem-loop primer and the mixture was placed at 95 °C for 5 min, 60 °C for 5 min, and on ice for 1 min. Subsequently, 4 μL of 5× first strand buffer, 2 μL of 0.1 M DTT, 1 μL of 10 mM dNTPs, 2 μL of diethyl pyrocarbonate (DEPC)-treated water and 1 μL of M-MLV reverse transcriptase (Thermo Fisher Scientific, Waltham, MA, USA) were added. Mixtures were incubated at 16 °C for 30 min, 42 °C for 30 min and 85 °C for 5 min. cDNA was serially diluted and used for PCR.

### 4.5. PCR and qRT-PCR Amplification of miRNAs

Initial PCR reactions were performed to confirm correct product amplification. Reactions consisted of 5 µL of cDNA (10^−1^), 5.5 µL of DEPC-treated water, 1 µL of 25 μM miRNA-specific forward primer, 1 μL of 25 μM universal reverse primer and 12.5 μL of 2× Taq FroggaMix (FroggaBio, Toronto, ON, Canada) [42]. Forward and universal primers used are presented in Table 6. An initial denaturing step at 95 °C for 5 min was performed, followed by 35 cycles at 95 °C for 15 s and at a gradient of temperatures for 1 min. Products were separated on a 2% agarose gel and visualized using a ChemiDoc MP system (Bio-Rad, Hercules, CA, USA). Products were then sequenced at the Université Laval (Quebec City, QC, Canada) and identities were confirmed using Basic Local Alignment Search Tool (BLAST).

The qRT-PCR reactions were performed by mixing 2.5 µL of diluted cDNA template with 0.5 µL of DEPC-treated water, 1 µL of 5 µM miRNA-specific forward primer, 1 µL of 5 µM universal reverse primer and 5 µL of 2× iTaq Universal SYBR Green Supermix (Bio-Rad, Hercules, CA, USA) prepared in triplicate in 96-well plates. Amplification protocol consisted of an initial denaturing step at 95 °C for 3 min, followed by 40 cycles at 95 °C for 15 s and optimal annealing temperature for each miRNAs (Table 6) for 30 s. Reactions were conducted on a CFX Connect Real-Time PCR Detection System (Bio-Rad, Hercules, CA, USA). Efficiencies were calculated for each primer pair with the same protocol as above in serial cDNA dilutions. NormFinder software (Aarhus University Hospital, Aarhus, Denmark) [43] revealed miR-1-3p as the most stably expressed miRNA amongst a group of transcripts in insects exposed to imidacloprid for 8 h as determined by qRT-PCR, and was used as reference transcript.

### 4.6. miRNA Transcript Targets Prediction

TargetScanFly 6.2 [44] and the fruit fly miRanda algorithm [45] target prediction tools were used to identify likely mRNA candidates regulated by the imidacloprid-associated miRNAs identified by high-throughput sequencing. The top five transcript targets linked with imidacloprid-modulated miRNAs that were available in both prediction tools were obtained. A list of miRNA targets was subsequently generated and functionally annotated with the Database for Annotation, Visualization and Integrated Discovery (DAVID) bioinformatics resources v6.8 [46]. Biological processes associated with these targets were generated.

### 4.7. Quantification and Statistics

The Cq values following qRT-PCR runs were obtained using the Bio-Rad CFX Manager software (Bio-Rad, Hercules, CA, USA). miRNA levels were normalized using miR-1-3p levels amplified from the same sample. The miRNA levels were measured using the 2^−ΔΔ*C*q^ method [47]. Ratios of normalized miRNA levels in control CPBs to average transcript expression in imidacloprid-treated CPBs were generated. Statistical differences between control and treated conditions were assessed with the Student’s *t*-test. For high-throughput sequencing, annotated sequences were normalized using the trimmed mean of M-values (TMM) function in edgeR Bioconductor [48], and log2 ratios depicting miRNA expression in insecticide-treated versus control CPBs were calculated. Bioconductor “limma” package, with linear model fit and empirical Bayes statistics, was used to calculate differential expression [49]. Results for miRNAs with average normalized expression (ANE) greater than 10, log2 fold-change higher than 0.3 and *p* < 0.05 were investigated.

## Figures and Tables

**Table 1:** Mapping statistics of reads in control versus imidacloprid-treated *L. decemlineata*.

Types of RNAs	Unique Reads Control/Treated	Percent Control/Treated	Read Count Control/Treated	Percent Control/Treated
Total reads	8,825,610/6,211,853	100%/100%	22,534,030/13,763,160	100%/100%
miRNAs	26,447/24,221	0.30%/0.39%	1,558,719/1,511,201	6.92%/10.98%
tRNAs	40,144/22,115	0.45%/0.36%	326,281/125,085	1.45%/0.91%
snRNAs	4705/2937	0.05%/0.05%	11,161/6302	0.05%/0.05%
snoRNAs	4735/3616	0.05%/0.06%	10,813/6910	0.05%/0.05%
rRNAs	102,238/64,189	1.16%/1.03%	619,781/172,969	2.75%/1.26%

**Table 2:** miRNAs strongly expressed in *L. decemlineata*.

miRNAs	miRNA Sequences	Normalized Expression
miR-14-3p	UCAGUCUUUUUCUCUCUCCUAU	88,717.57
miR-8-3p	UAAUACUGUCAGGUAAAGAUGUC	73,723.79
miR-276-3p	UAGGAACUUCAUACCGUGCUCU	54,681.67
miR-317-3p	UGAACACAGCUGGUGGUAUCUCAGU	39,445.82
miR-1-3p	UGGAAUGUAAAGAAGUAUGGAG	16,976.97
miR-2a-3p	UAUCACAGCCAGCUUUGAUGAGC	16,697.46
miR-281-5p	AAGAGAGCUAUCCGUCGACAGU	16,606.36
miR-1175-3p	UGAGAUUCAACUCCUCCAUCUC	14,289.62
miR-13b-3p	UAUCACAGCCAUUUUGACGAGU	14,017.42
bantam-3p	UGAGAUCAUUGUGAAAGCUGAUU	13,166.75

Presented average normalized expressions are from all samples characterized by high-throughput sequencing.

**Table 3:** Differential expression of 33 miRNAs following imidacloprid treatment in *L. decemlineata*.

miRNAs	miRNA Sequences	Normalized Expression Treated/Control		Log2 Fold-Change Treated/Control
miR-iab-8-3p	AGGAUACAUUCAGUAUACG	27.77	53.71	0.95
miR-252a-3p	CCUGCUGCUCAAGUGCUUAUC	12.40	21.64	0.80
miR-282-5p	UAGCCUCUCCUAGGCUUUGUCU	13.23	20.32	0.62
miR-iab-8-5p	UUACGUAUACUGAAGGUAUACCGGAC	30.54	44.06	0.53
miR-1000-5p	AUAUUGUCCUGUCACAGC	63.29	86.10	0.44
miR-3849-5p	UGACAUUUUAACCAUAGUGCU	59.97	81.16	0.44
miR-193-3p	UACUGGCCUGUUAAGUCCCAAGU	57.31	77.14	0.43
miR-7-3p	CAAGGAAUCACUAAUCAUCCCAC	36.79	49.25	0.42
miR-124-3p	UAAGGCACGCGGUGAAUGCCAAG	183.64	239.11	0.38
miR-970-3p	UCAUAAGACACACGCGGCUAU	337.13	427.79	0.34
miR-276-5p	AGCGAGGUAUAGAGUUCCUACGUG	2120.16	2672.97	0.33
let-7-5p	UGAGGUAGUAGGUUGUAUAG	4613.21	5776.29	0.32
miR-1-3p	UGGAAUGUAAAGAAGUAUGGAG	15,177.88	18,776.07	0.31
miR-133-3p	UUGGUCCCCUUCAACCAGCUGU	1771.68	2187.69	0.30
miR-263b-5p	CUUGGCACUGGAAGAAUUCAC	95.91	76.77	−0.32
miR-281-5p	AAGAGAGCUAUCCGUCGACAGU	18,510.91	14,701.80	−0.33
miR-2944c-3p	UAUCACAGCCAGUAGUUACC	4468.29	3543.74	−0.33
miR-1175-5p	AAGUGGAGCAGUGGUCUCUUCAC	286.32	224.91	−0.35
miR-92a-3p	AUUGCACUAGUCCCGGCCUA	62.52	48.60	−0.36
miR-305-5p	AUUGUACUUCAUCAGGUGCUC	7239.92	5605.19	−0.37
miR-iab-4-3p	CGGUAUACCUUCAGUAUACGUAAC	24.56	18.65	−0.40
miR-927a-5p	UUUAGAAUUCCUACGCUUUA	20.10	14.98	−0.42
miR-9c-5p	UCUUUGGUGAUCUAGCCGUGUG	405.18	297.72	−0.44
miR-34-3p	CGACCACUAUCCAUACUCCCUCC	29.11	21.28	−0.45
miR-316-5p	UGUCUUUUUCCGCUUUGCUGC	11,436.58	8348.15	−0.45
miR-998-3p	UAGCACCAUGGGAUUCAGCUCA	87.90	59.03	−0.57
miR-100-5p	AACCCGUAGAUCCGAACUUGUGGG	5341.40	3528.46	−0.60
miR-750-3p	CCAGAUCUAACUCUUCCAUAUGACG	6089.67	3795.84	−0.68
miR-995-3p	UAGCACCACAUGAUUCAGCUUACG	551.70	340.13	−0.70
miR-2796-5p	AGGGGUUUCUUUCGGCCUCCAGCG	41.19	24.70	−0.74
miR-92a-5p	AGUCCGUGAUGCGUGACAAUAU	197.04	117.23	−0.75
miR-315-5p	UUUUGAUUGUUGCUCAGAAAGC	21.80	12.80	−0.77
miR-989-3p	UGUGAUGUGACGUAGUGG	4764.90	2748.54	−0.79

**Table 4:** Target prediction of select *L. decemlineata* miRNAs with TargetScanFly and miRanda.

miRNAs	TargetScanFly Targets	MiRanda Targets
miR-282-5p	Ero1L, Fus, CG14435, CG9515, CcapR	Meso18E, Rogdi, Resilin, Nkd, Kraken
miR-1000-5p	CG34355, Nplp1, CG10804, Net, CG13384	Nplp1, CG10804, Kon, CG13384, Net
miR-193-3p	P38b, Cp7Fb, Ana, CG11041, CG11313	CG34394, RYBP, CG6707, Pb, CG32736
miR-124-3p	Sinu, Gli, CG12977, Pk92B, Axs	Pk92B, CG14299, Sinu, Gli, Cp110
miR-970-3p	Btsz, CG15097, Ru, Ace, Rab6	CG15097, Ubc-E2H, CG32372, CG42256, StmA
let-7-5p	Ab, CG18265, A3-3, Apt, Cpr49Aa	Ab, CG5026, CG34118, CG9548, Slam
miR-1-3p	CG4297, Tub, CG30457, Hmu, CG6490	Par-6, CG18542, Pen, CG5053, Pdm2
miR-133-3p	Fili, CG17193, CG2774, CG33324, CG9541	CG30409, SkpA, Wts, Pde1c, CG32351
miR-263b-5p	Qkr54B, Bx, Wls, CG32062, CG34339	CG2371, Wls, Qkr54B, Bx, GATAe
miR-92a-3p	Sha, Khc-73, CrebA, CG4297, Tusp	CrebA, Cpr50Ca, CG8128, CG3077, CG14274
miR-305-5p	CG33174, CG11997, CG3287, Gr98d, Nerfin-1	CG3287, CG31855, Mi-2, NfI, Eya
miR-927a-5p	Gprs, CG32245, Kr-h1, CG8485, Growl	CG8485, EcR, CG9850, Aef1, Sfl
miR-9c-5p	Nerfin-1, Rbp9, CG11206, CG32333, Bru-2	Sinu, CadN, CG5746, CG11206, CG9426
miR-316-5p	Hr39, Rbp9, CG32121, Numb, Bsg25D	Nkd, Numb, Aret, Cib, RdgC
miR-100-5p	E(Pc), Gogo, CG17985, CG31772, DopR2	CG3630, CG10979, Gogo, Pc, CG13326
miR-315-5p	Eip93F, CG15465, CG32333, CG32137, CG32206	CG12424, CG14989, CG32137, Rtnl1, CG34126
miR-989-3p	Tankyrase, Fal, Lac, PGRP-SD, Qkr54B	CG12772, Chrw, CG34449, Nedd4, Kni

**Table 5:** Biological processes predicted to be modulated by select miRNAs with varying expression levels in imidacloprid-treated *L. decemlineata*.

GO Biological Process	Genes Targeted	miRNAs	*p*-Value
Sensory perception of pain	13	11	6.0 × 10^−3^
Regulation of transcription, DNA-templated	12	10	7.2 × 10^−4^
Transcription, DNA-templated	11	9	1.6 × 10^−3^
Regulation of glucose metabolic process	9	7	1.5 × 10^−4^
Positive regulation of transcription RNA pol II promoter	8	7	3.8 × 10^−3^
Border follicle cell migration	6	6	5.7 × 10^−3^
Axon guidance	6	5	2.3 × 10^−2^
Imaginal disc-derived wing morphogenesis	6	5	4.1 × 10^−2^
Muscle organ development	5	6	5.9 × 10^−3^
Negative regulation of transcription, DNA-templated	5	5	2.1 × 10^−2^
Negative regulation of transcription RNA pol II promoter	5	5	4.1 × 10^−2^

**Table 6:** Sequences, efficiencies, and optimal melting temperatures of primers used in this study.

Primer	Sequence	Eff.	Temp.
miR-1-3p	5′-ACACTCCAGCTGGGTGGAATGTAAAGAAGTA-3′	92.0%	62.5 °C
	5′-CTCACAGTACGTTGGTATCCTTGTGATGTTCGATGCCATATTGTACTGTGAGCTCCATAC-3′		
miR-7-5p	5′-ACACTCCAGCTGGGTGGAAGACTAGTGAT-3′	96.0%	60.0 °C
	5′-CTCACAGTACGTTGGTATCCTTGTGATGTTCGATGCCATATTGTACTGTGAGCACAACAA-3′		
miR-9a-5p	5′-ACACTCCAGCTGGGTCTTTGGTTATCTAG-3′	101.4%	58.8 °C
	5′-CTCACAGTACGTTGGTATCCTTGTGATGTTCGATGCCATATTGTACTGTGAGTCATACAG-3′		
miR-12-5p	5′-ACACTCCAGCTGGGTGAGTATTACATCAGGT-3′	95.6%	64.5 °C
	5′-CTCACAGTACGTTGGTATCCTTGTGATGTTCGATGCCATATTGTACTGTGAGCAGTACCT-3′		
miR-92a-5p	5′-ACACTCCAGCTGGGAGTCCGTGATGCGTGAC-3′	88.5%	56.5°C
	5′-CTCACAGTACGTTGGTATCCTTGTGATGTTCGATGCCATATTGTACTGTGAGATATTGTC-3′		
miR-133-3p	5′-ACACTCCAGCTGGGTTGGTCCCCTTCAACCA-3′	84.3%	64.5 °C
	5′-CTCACAGTACGTTGGTATCCTTGTGATGTTCGATGCCATATTGTACTGTGAG-3′		
miR-305-5p	5′-ACACTCCAGCTGGGATTGTACTTCATCAGGT-3′	91.3%	63.4 °C
	5′-CTCACAGTACGTTGGTATCCTTGTGATGTTCGATGCCATATTGTACTGTGAGGAGCACCT-3′		
miR-927a-5p	5′-ACACTCCAGCTGGGTTTAGAATTCCTACGCT-3′	105.3%	60.9 °C
	5′-CTCACAGTACGTTGGTATCCTTGTGATGTTCGATGCCATATTGTACTGTGAGTAAAGCGT-3′		
miR-989-3p	5′-ACACTCCAGCTGGGTGTGATGTGACGTAGTG-3′	99.5%	56.9 °C
	5′-CTCACAGTACGTTGGTATCCTTGTGATGTTCGATGCCATATTGTACTGTGAGCCACTACG-3′		
Universal	5′-CTCACAGTACGTTGGTATCCTTGTG-3′	-	-

Top and bottom sequences represent forward and stem-loop primers, respectively. Abbreviations: Efficiency (Eff.); Temperature (Temp.).
